# Survey and Diversity of *Grapevine Pinot gris virus* in Algeria and Comprehensive High-Throughput Small RNA Sequencing Analysis of Two Isolates from *Vitis vinifera* cv. Sabel Revealing High Viral Diversity

**DOI:** 10.3390/genes11091110

**Published:** 2020-09-22

**Authors:** Aleš Eichmeier, Eliška Peňázová, Jana Čechová, Akila Berraf-Tebbal

**Affiliations:** Faculty of Horticulture, Mendeleum-Institute of Genetics, Mendel University in Brno, Valticka 334, 69144 Lednice, Czech Republic; penazova.e@gmail.com (E.P.); jana.cechova@mendelu.cz (J.Č.); berraf.a@hotmail.fr (A.B.-T.)

**Keywords:** high-throughput small RNA sequencing, grapevine, RT-PCR, *Grapevine Pinot gris virus*, RdRp

## Abstract

*Grapevine Pinot gris virus* (GPGV) is a putative causal agent of grapevine leaf mottling and deformation disease that has been reported worldwide throughout the grapevine-growing regions. Fifty-four grapevines collected from five Algerian grapevine-growing regions were tested for the presence of GPGV in phloem tissues. Eight of the tested grapevines were infected by GPGV. Viromes of two selected *Vitis vinifera* cv. Sabel grapevines infected by GPGV and showing virus-like symptoms were analyzed by small RNA sequencing. Phylogenetic analyses of the partial coding sequence (cds) of the RNA-dependent RNA polymerase (RdRp) domain showed that all Algerian GPGV isolates were grouped with some already-described asymptomatic isolates. This study provides the first survey of the occurrence of GPGV in Algeria. Moreover, *Grapevine fleck virus*, *Grapevine rupestris stem pitting-associated virus*, *Grapevine virus B*, *Grapevine rupestris vein feathering virus*, *Hop stunt viroid* and *Grapevine yellow speckle viroid 1* were detected in Algeria for the first time.

## 1. Introduction

*Grapevine Pinot gris virus* (GPGV) is a member of the genus *Trichovirus* in the family Betaflexiviridae. The virus was initially described in the Italian grapevine cv. Pinot gris in which it causes grapevine leaf mottling and deformation disease (GLMD) [1,2]. GPGV is widespread in many wine-producing countries around the world [3], and was more recently detected in Canada [4], Armenia [5], Australia [6], Brazil [7], Chile [8] and Pakistan [9].

It is not easy to assess the economic impact of GPGV because many strains do not provoke GLMD symptoms [2,3,10]. Several studies in Italy have monitored the production and growth parameters of GLMD-affected/GPGV-infected vineyards planted with varieties that are expected to be more sensitive to GPGV infection [2,11,12]. Observations performed in Prosecco vines in Veneto, northeast Italy, showed a reduction in bunch quality and led to the removal of GLMD-affected plants and subsequent economic loss [13]. Similar studies in other wine-growing countries are required to investigate the impact of specific GPGV strains, both alone and in combination with other viruses, on individual grapevines by accurately measuring fruit yield, fruit quality, vegetative growth and grapevine sustainability. Algeria has 75,000 ha of vineyards, and 65% of this area is planted with table grapes, 34.4% with wine grapes, 0.5% with mother plants and only 0.1% with grapes for the production of raisins [14,15].

The genomic RNA of GPGV consists of three open reading frames (ORFs). ORF1 represents 1865 amino acids (aa) (214 kDa) and encodes replicase-associated proteins, methyltransferase (44–333 aa), helicase (1040–1277 aa) and RNA-dependent RNA polymerase (RdRp) (1447–1797 aa). ORF2 encodes a 376 aa (42 kDa) polypeptide homologous to a movement protein, and ORF3 encodes the 195 aa (22 kDa) putative coat protein [1]. According to Hily et al. [16], ORF1 of most isolates is composed of 1855 aa, and only the Goldfinger isolate has a different size. Studies of the genome expression of *Apple chlorotic leaf spot virus* [17] suggest that the GPGV strategy of RNA translation and replication likely relies on polyprotein processing of subgenomic RNAs.

The presence of sequence variations in GPGV isolates remains unknown worldwide. There have been a few studies on the variability of the GPGV genome, and they have suggested that the genome is genetically diverse and consists of numerous haplotypes [2,5,12,18,19]. Recently, Hily et al. [16] published genetic diversity analyses of 100 new complete or near-complete GPGV genomic sequences.

The grapevine virome is not well described in Algeria. There have been surveys of *Grapevine leafroll-associated virus-1* [20], *Grapevine leafroll-associated virus-2* [21] and *Grapevine leafroll-associated virus-3* [22]; however, a comprehensive virological study of grapevines has not been performed in Algerian vineyards. Therefore, we introduced high-throughput small RNA sequencing analysis as a tool for obtaining general and comprehensive information about viral diversity in selected grapevines.

## 2. Materials and Methods

### 2.1. Sampling and Collection of Viral Isolates

In June, 2018, field surveys were conducted in nine vineyards located in five wine-growing regions in Algeria: Médéa (Benchicao), Tipaza (Hamr El Ain), Tipaza (Hadjout), Boumerdes and Alger (Table 1). Fifty-four grapevines showing leaf deformation were collected from the vineyards, which were older than 10 years. The wooden internodes were cut from the grapevines in situ and were packed and transferred to Mendelem Laboratory, Department of Genetics, Faculty of Horticulture, MENDELU, Czech Republic. A total of 54 grapevines were tested for the presence of GPGV.

### 2.2. RNA Extraction and RT-PCR Detection of GPGV and Sequencing of PCR Amplicons

RNA was extracted directly from 1 g of scraped phloem using the Spectrum Plant Total RNA Kit (Sigma–Aldrich, St. Louis, MO, USA). RNA was retrotranscribed to cDNA as described by Eichmeier et al. [23]. Subsequently, PCR was performed utilizing GoTaq^®^ G2 Flexi DNA Polymerase (Promega, Madison, WI, USA). To broaden our knowledge of GPGV occurrence in Algeria, we used PCR assays targeting the RdRp domain of the replicase gene [2]. We used this assay because we previously found that detection of the GPGV RdRp domain was more effective than the assay targeting the MP/CP gene sequences of this virus [24]. PCR amplicons were sequenced by Sanger sequencing as described by Eichmeier et al. [23].

### 2.3. Phylogenic Analysis of Partial RdRp Domain

To determine the phylogeny that can be linked to the symptomatic manifestations of the GPGV isolates, sequences available in GenBank and sequences from our previous phylogenetic studies were used, showed the symptomatic manifestations of GPGV infection causing GLMD. We placed the new sequences of the Algerian RdRp domains into the phylogenetic pattern published by Eichmeier et al. [3] using the same algorithms and parameters.

### 2.4. Small RNA Sequencing

The same extracted total RNA obtained for RT-PCR was used for library preparation. A small RNA library was constructed using the TruSeq Small RNA Library Preparation Kit (Illumina, San Diego, CA, USA) and purification and quality control were done as described by Eichmeier et al. [25]. For the sequencing run, the final pooled library of small RNAs consisted of two samples: AL_41 was labeled with index 9 (GATCAG) and AL_42 was labeled with index 10 (TAGCTT). The library was sequenced with the MiniSeq instrument (Illumina) using the MiniSeq High Output Reagent Kit (75-cycles) (Illumina) providing 36-nucleotide-long reads.

### 2.5. Sequence Data Analysis

Quality control was performed by FastQC-0.10.1 software [26]. A FASTX-Toolkit Clipper (http://hannonlab.cshl.edu/fastx_toolkit/), specifying the Q30 parameter, was used to remove the adaptors (TGGAATTC), and sequences shorter than 15 nucleotides were discarded. Contigs of individual reads were assembled de novo using Velvet-1.2.10 [27] with k-mer of 15. The obtained contigs were screened for homology to identify the viruses by Nucleotide-Nucleotide BLAST 2.2.31+ in the unix environment, using the newest version of viral.1.1 genomic database of NCBI. A threshold e-value was set as 10^−5^ [18]. The same list of reference sequences was used for mapping of the reads, as described by Eichmeier et al. [28]. Then, sequencing reads were mapped using CLC Genomics Workbench 6.5.1 (CLC Bio, Aarhus, Denmark) on the reference sequences with the following parameters: mismatch cost = 2 (the cost of a mismatch between the read and the reference sequence); insertion cost = 3 (the cost of an insertion in the read causing a gap in the reference sequence), and deletion cost = 3 (the cost of having a gap in the read). These parameters were used for global alignment, and the reads were matched randomly.

### 2.6. Determination of Presence of Grapevine Viruses 

The presence of the viruses was determined based on the consensus of the results regarding the BLASTn contigs and mapped reads in the references. In contrast to our previous studies, where the sequencing depth was greater [18,28], in this study, we acquired low numbers of reads, and we did not establish the genome coverage and sequencing depth threshold to clearly determine the presence of the virus. In each case in which viral reads were obtained, we performed the specific RT-PCR protocols listed in Section 3.3.

## 3. Results

### 3.1. Results of GPGV RT-PCR Detection

Fifty-four grapevines were tested for the presence of GPGV in phloem tissues. We detected eight GPGV-positive grapevines (Table 2). We identified one positive sample of cv. Hmer Bouameur from the Médéa region, one positive sample of cv. Alphonse-Lavallée from the region of Alger and six positive samples from the remaining grapevines (from a total of 12) of cv. Sabel from the Boumerdes region. Each of the obtained PCR amplicons was sequenced, and the sequences were deposited in GenBank under Acc. Nos. MT832147–MT832154.

**Figure 1:**
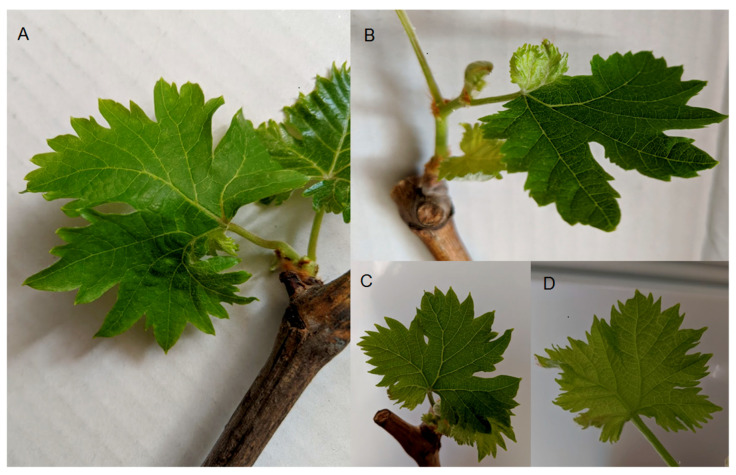
Symptoms frequently observed in GPGV-positive cv. Sabel sampled in the Boumerdes region. (**A**) Deformation of the leaf basis (AL_41); (**B**) greening of the leaf basis (AL_41); (**C**,**D**) leaf deformation (AL_42).

Grapevines in the Boumerdes region showed grapevine leaf deformation (Figure 1) but did not show typical grapevine leaf mottling and deformation symptoms as described previously [1,2]. Based on the strongest observed leaf deformation symptoms, the grapevines AL_41 and AL_42 were selected for further analysis using small RNA sequencing to reveal the virome of the selected grapevines.

### 3.2. Phylogenetic Analysis

**Figure 2:**
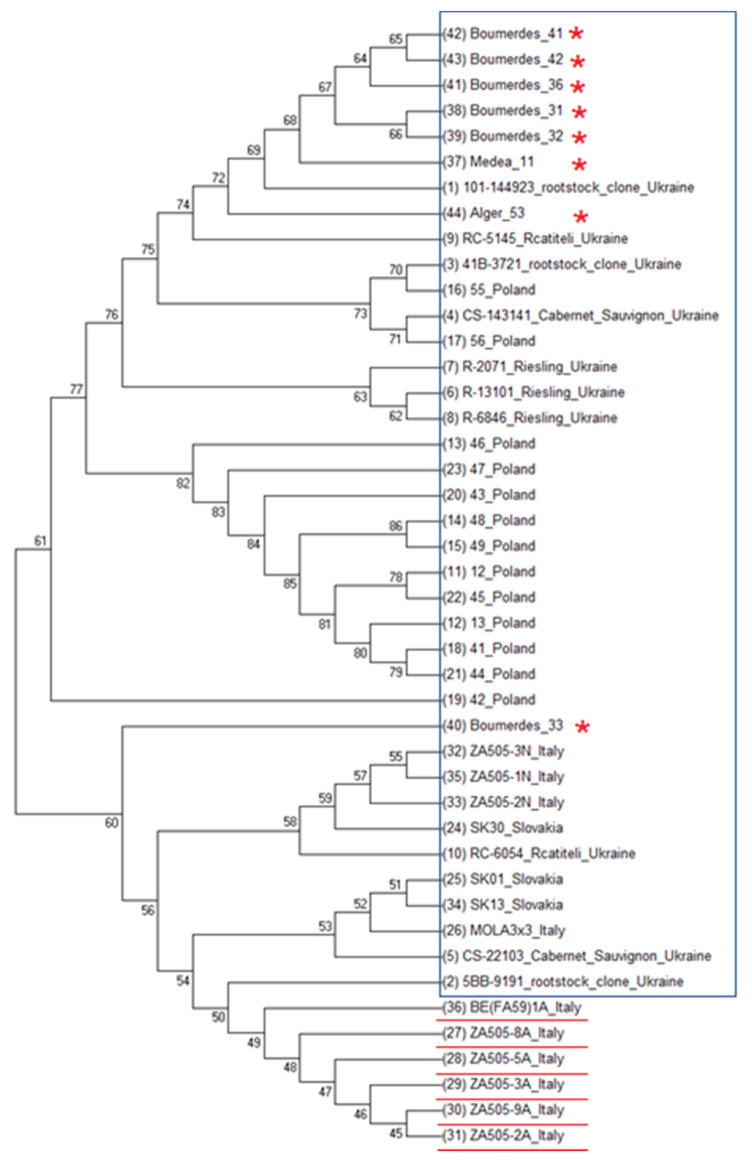
Molecular phylogenetic analysis of the RdRp domain sequences using the maximum likelihood method based on the Tamura–Nei model [29]. Algerian GPGV isolates and the isolates determined to be symptomatic and asymptomatic were included [2,3,24]. The isolates in the box did not cause typical GPGV symptoms. The cladogram was constructed using MEGA 7 [30], Muscle [31] and the UPGMB clustering method. Node IDs (sorting of the sequences) corresponded to the tabular description of the timetree in the text editor. All isolate sequences and individual nodes had IDs (see Supplementary Material Table S1). The isolates in the box did not cause typical grapevine leaf mottling and deformation (GLMD) symptoms, the isolates highlighted by red asterisks were obtained in this study, and the underlined isolates caused GLMD symptoms.

Molecular phylogenetic analysis based on the RdRp domain sequences compared 44 sequences from Italy and Slovakia [2], Poland [24] and Ukraine [3], and 8 sequences of Algerian GPGV isolates (Figure 2). This pattern was used on the basis of the previous studies showing the symptomatic/asymptomatic manifestations of the GPGV isolates in the plants. Algerian isolates from the Bourmedes and Médéa regions were the closest to each other and clustered in the upper part of the phylogenetic tree. The isolates of the Bourmedes region created a separate group except for one isolate that was clustered in a separate space between the Polish and asymptomatic Italian isolates. Isolate Alger_53 was clustered among the Ukrainian GPGV isolates. The tree indicates that the Algerian GPGV isolates were probably not the causal agents of the observed symptoms. None of the Algerian isolates showed the symptoms described by Giampetruzzi et al. [1].

### 3.3. Detection of the Viruses and Viroids by Small RNA Sequencing

**Figure 3:**
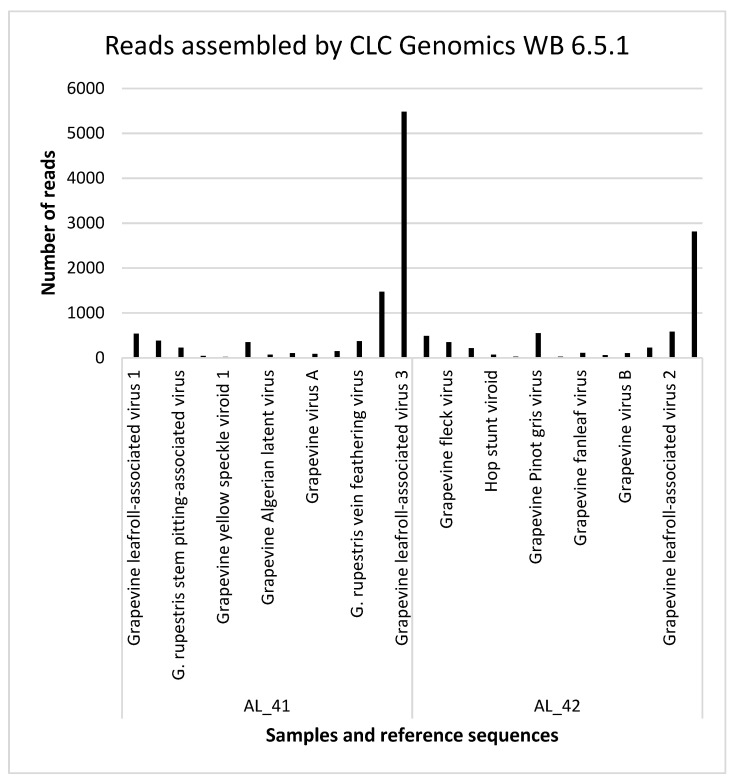
Numbers of reads mapped on the reference viral and viroid sequences. CLC Genomics WB 6.5.1 (CLC Bio, Aarhus, Denmark) was used to plot the data.

Sequenced libraries represented the sRNA populations extracted from the two selected grapevines: AL_41 and AL_42 of cv. Sabel from the Bourmedes region. The libraries were sequenced by the sequencing by synthesis (SBS) approach and contained 1,588,329 reads (AL_41) and 1,604,641 reads (AL_42). De novo assembly of the sequenced reads and a BLAST search for homologies of the obtained contigs identified eight viruses and two viroids in two grapevines (Table 3 and Figure 3). Two viroids were present in both tested grapevines, and *Grapevine virus B* and *Grapevine rupestris vein feathering virus* were detected only in AL_41. The results of small RNA sequencing were verified by RT-PCR detection, which indicated that all the detected viruses and viroids were indeed present in the tested materials, thus achieving verification by at least two methods (see Discussion and Table 3). The BLAST/NCBI of the AL_41 GPGV of complete cds RdRp showed the highest similarity with an Italian GPGV isolate (Acc. No. MH087455, isolate fvg-Is14), which was isolated and sequenced from the plants showing symptoms of GLMD: 4029/5437 (74.10%) nucleotide identity and 1714/1859 (92%) amino acid identity. The complete cds RdRp of AL_42 had the highest nucleotide identity (3697/4974, 74%) and amino acid identity (1540/1699, 91%) with a Californian isolate (Acc. No. MK514520; isolate S103).

### 3.4. Description of Detected Viruses

*Grapevine fleck virus* (GFkV), genus *Maculavirus*, family Tymoviridae, was detected in both grapevines. A total of 382 reads were assembled for grapevine AL_41 (genome coverage 51%) and 348 reads for AL_42 (genome coverage 53%). GFkV causes latent infections in *Vitis vinifera* cultivars, but induces specific foliar symptoms in the indicator host, *Vitis rupestris*. The foliar symptoms include clearing of the veinlets in young leaves. Those symptoms were not observed.

*Grapevine rupestris stem pitting-associated virus* (GRSPaV), belonging to the genus *Foveavirus*, family Betaflexiviridae, was detected in both AL_41 and AL_42 grapevines. A total of 227 reads were assembled by CLC Genomics WB 6.5.1 (genome coverage 17%) for AL_41 and 216 reads (genome coverage 19%) for AL_42. GRSPaV infection was confirmed by RT-PCR in both isolates. Symptoms of GRSPaV, modified wood (pitting), were not observed.

*Hop stunt viroid* and *Grapevine yellow speckle viroid 1* were detected in both grapevines. These viroids are reported to occur worldwide in wine-producing countries as latent viroids in grapevines. Their economic impact on grapevines has not been reported.

GPGV was reliably detected in both grapevines, with a genome coverage ranging from 74% and 349 reads (AL_41) to 89% and 549 reads (AL_42).

*Grapevine virus B* (GVB), genus *Vitivirus*, family Betaflexiviridae, was detected in plant AL_41, and 149 reads were assembled by CLC Genomics WB 6.5.1 and genome coverage was only 3%. RT-PCR clearly confirmed the presence of the virus. The virus is associated with rugose wood symptoms in grapevines and the symptoms were not observed in grapevine AL_41.

*Grapevine rupestris vein feathering virus* (GRVFV), genus *Marafivirus*, family Tymoviridae, was detected in plant AL_41, 371 reads were assembled and genome coverage was 13%. RT-PCR confirmed the presence of the virus. The virus causes asteroid-like symptoms that were not observed on grapevine AL_41.

*Grapevine leafroll-associated virus 2* (GLRaV-2), genus *Ampelovirus*, family Closteroviridae, was detected in plants AL_41 (1473 assembled reads, genome coverage 59%) and AL_42 (584 assembled reads, genome coverage 29%). The leafroll symptoms were not observed.

*Grapevine leafroll-associated virus 3* (GLRaV-3), genus *Ampelovirus*, family Closteroviridae, was detected in plants AL_41 (5479 assembled reads, genome coverage 79%) and AL_42 (2809 assembled reads, genome coverage 43%). The virus had the greatest number of reads among all of the viruses. The leafroll symptoms were not observed.

## 4. Discussion

The survey of GPGV was performed according to the observed symptoms in 54 grapevines in five Algerian regions. In Algeria, there is no clear evidence of the presence of grapevine viruses in the vineyards, except for the surveys of GLRaV-1 [20], GLRaV-2 [21] and GLRaV-3 [22].

We detected GPGV in 8 out of 54 grapevines (15% of the tested grapevines). We observed the symptoms as described in the Results, but none of the symptoms (GLMD) appeared to be linked with GPGV infection. This observation is supported by the phylogenetic analysis of the partial RdRp domain as initially described by Saldarelli et al. [2]. The etiology of the symptoms presented in Figure 1 seems to be unknown, even when revealing the viruses by high-throughput small RNA sequencing. The phylogenetic study demonstrated that the Algerian GPGV isolates were genetically similar. The geographic uniqueness of GPGV isolates was confirmed by Eichmeier et al. [3,5,10,24]. Given the similarities between GLMD and boron deficiency symptoms in grapevines, Buoso et al. [41] suggested that GPGV interferes in boron homeostasis. However, two studies suggested that the association between the symptoms and the presence of the virus is unclear [2,42]. Tarquini et al. [43] have not demonstrated any differences in the ultrastructural cytopathy induced by GPGV in symptomatic and asymptomatic plants. The absence of a strict and complete association between the GPGV isolate clustering and symptom expression is in agreement with the strong similarity of the genome sequences of GPGV isolates collected from symptomatic and asymptomatic grapevines reported in various studies [4,10,44]. We found that the highest similarity of the complete cds RdRp domain of AL_41 was detected in an Italian GPGV isolate, fvg-Is14, which caused symptoms of GLMD [45]. In the present study, there was no evidence of boron deficiency, which may be a key factor required for the manifestation of GLMD symptoms in combination with GPGV infection of the grapevines.

In this study, we detected GLRaV-2 and GLRaV-3 in agreement with the studies of Lehad et al. [21,22]. According to high-throughput small RNA sequencing, GLRaV-1 should be present; however, RT-PCR revealed that the samples were not clearly positive, and detection of GLRaV-1 alone in AL_41 did not clearly identify the PCR product [33] that could not be clearly established as virus positive. The negative results of the RT-PCR detection of GLRaV-1 may have been caused by the fact that high-throughput small RNA sequencing can be more sensitive than RT-PCR.

Lehad et al. [20] reported that GLRaV-1 was present in 5.4% of 484 grapevines in Algeria. GLRaV-2 was detected by Lehad et al. [21] in 15.8% of Algerian vineyards, with 584 samples tested. GLRaV-3 was surveyed by Lehad et al. [22] and was detected in 44% of 484 samples. Detection of these viruses of the GLRaV group was verified in two samples, AL_41 and AL_42, in the present study, in agreement with the surveys of Lehad et al. [20,21,22], indicating that these viruses may be widespread in Algeria. However, we did not observe the typical leafroll symptoms.

To the best of our knowledge, this study is the first to report the detection of Grapevine fleck virus, Grapevine rupestris stem pitting-associated virus, Grapevine virus B, and Grapevine rupestris vein feathering virus, and viroids Hop stunt viroid and Grapevine yellow speckle viroid 1 in Algeria.

## 5. Conclusions

The outcomes of this study provide novel insights into the presence of GPGV in grapevines in Algeria. The results revealed that GPGV did not show typical symptomatic behavior (GMLD) in eight out of fifty-four sampled grapevines. The GPGV isolates were mostly clustered in a separate group, showing that GPGV in Algeria consists of local strains. Additionally, based on high-throughput small RNA sequencing, the two selected cv. Sabel grapevines showed infection with nine viruses and viroids. *Grapevine fleck virus*, *Grapevine rupestris stem pitting-associated virus*, *Grapevine virus B*, *Grapevine rupestris vein feathering virus* and viroids *Hop stunt viroid* and *Grapevine yellow speckle viroid 1* are reported in Algeria for the first time.

## Figures and Tables

**Table 1:** List of Algerian regions for grapevine sampling and the number of sampled grapevines.

Sampling Regions	Altitude (m)	GPS Coordinates	Number of Plants	Cultivar
Medea (Benchicao)	1006	36°12′4″ N 2°49′58″ E	12	Hmer Bouameur
Tipaza (Ahmer El Aïn)	85	36°23′60″ N 2°51′35″ E	12	Carignan
Tipaza (Hadjout)	72	36°31′36″ N 2°24′7″ E	6	Alphonse-Lavallée
Boumerdes (Bordj Menaiel)	52	36°45′14″ N 3°40′28″ E	12	Sabel
Alger (Ain Benian)	0	36°47′40″ N 2°55′53″ E	12	Cardinal, Alphonse-Lavallée

**Table 2:** Results of the *Grapevine Pinot gris virus* (GPGV) detection.

Sample No.	Region	Cultivar	RdRp Amplification	Partial RdRp, GenBank Acc. No.	Full-Length RdRp, GenBank Acc. No.
AL_1	Médéa (Benchicao)	Hmer Bouameur	−		
AL_2	Médéa (Benchicao)	Hmer Bouameur	−		
AL_3	Médéa (Benchicao)	Hmer Bouameur	−		
AL_4	Médéa (Benchicao)	Hmer Bouameur	−		
AL_5	Médéa (Benchicao)	Hmer Bouameur	−		
AL_6	Médéa (Benchicao)	Hmer Bouameur	−		
AL_7	Médéa (Benchicao)	Hmer Bouameur	−		
AL_8	Médéa (Benchicao)	Hmer Bouameur	−		
AL_9	Médéa (Benchicao)	Hmer Bouameur	−		
AL_10	Médéa (Benchicao)	Hmer Bouameur	−		
AL_11	Médéa (Benchicao)	Hmer Bouameur	+	MT832147	
AL_12	Médéa (Benchicao)	Hmer Bouameur	−		
AL_13	Tipaza (Hamr El Ain)	Carignan	−		
AL_14	Tipaza (Hamr El Ain)	Carignan	−		
AL_15	Tipaza (Hamr El Ain)	Carignan	−		
AL_16	Tipaza (Hamr El Ain)	Carignan	−		
AL_17	Tipaza (Hamr El Ain)	Carignan	−		
AL_18	Tipaza (Hamr El Ain)	Carignan	−		
AL_19	Tipaza (Hamr El Ain)	Carignan	−		
AL_20	Tipaza (Hamr El Ain)	Carignan	−		
AL_21	Tipaza (Hamr El Ain)	Carignan	−		
AL_22	Tipaza (Hamr El Ain)	Carignan	−		
AL_23	Tipaza (Hamr El Ain)	Carignan	−		
AL_24	Tipaza (Hamr El Ain)	Carignan	−		
AL_25	Tipaza (Hadjout)	Alphonse-Lavallée	−		
AL_26	Tipaza (Hadjout)	Alphonse-Lavallée	−		
AL_27	Tipaza (Hadjout)	Alphonse-Lavallée	−		
AL_28	Tipaza (Hadjout)	Alphonse-Lavallée	−		
AL_29	Tipaza (Hadjout)	Alphonse-Lavallée	−		
AL_30	Tipaza (Hadjout)	Alphonse-Lavallée	−		
AL_31	Boumerdes	Sabel	+	MT832148	
AL_32	Boumerdes	Sabel	+	MT832149	
AL_33	Boumerdes	Sabel	+	MT832150	
AL_34	Boumerdes	Sabel	−		
AL_35	Boumerdes	Sabel	−		
AL_36	Boumerdes	Sabel	+	MT832151	
AL_37	Boumerdes	Sabel	−		
AL_38	Boumerdes	Sabel	−		
AL_39	Boumerdes	Sabel	−		
AL_40	Boumerdes	Sabel	−		
AL_41	Boumerdes	Sabel	+	MT832152	MT843110
AL_42	Boumerdes	Sabel	+	MT832153	MT843111
AL_43	Alger	Cardinal	−		
AL_44	Alger	Cardinal	−		
AL_45	Alger	Cardinal	−		
AL_46	Alger	Cardinal	−		
AL_47	Alger	Cardinal	−		
AL_48	Alger	Cardinal	−		
AL_49	Alger	Alphonse-Lavallée	−		
AL_50	Alger	Alphonse-Lavallée	−		
AL_51	Alger	Alphonse-Lavallée	−		
AL_52	Alger	Alphonse-Lavallée	−		
AL_53	Alger	Alphonse-Lavallée	+	MT832154	
AL_54	Alger	Alphonse-Lavallée	−		

RT-PCR was performed using primers designed by Saldarelli et al. [2]. The sequences of positive samples are available under listed Acc. Nos. The last column contains the sequences of the full-length RNA-dependent RNA polymerase (RdRp) domain genes of the two selected isolates (Al_41 and AL_42) that were obtained via small RNA sequencing. +, positive detection; −, negative detection.

**Table 3:** Results of small RNA sequencing and RT-PCR analysis.

Grapevine	Reference Accession Nr.	Virus	Contigs (Velvet, k-15) Identified by Blast (1E-5)	Reads Assembled by CLC Genomics WB 6.5.1	Average Seq Depth	Genome Coverage	RT-PCR Result the Protocol Reference
AL_41	NC_016509.1	Grapevine leafroll-associated virus 1	0	538	0	13%	− (Kominek et al., 2005) [32], − (Gambino a Gribaudo 2006) [33]
	NC_003347.1	Grapevine fleck virus	59	382	0.22	51%	+ (Sabanadzovic et al., 1996) [34]
	NC_001948.1	G. rupestris stem pitting-associated virus	1	227	0.03	17%	+ (Terlizzi et al., 2011) [35]
	NC_001351.1	Hop stunt viroid	0	40	0.15	26%	+ (Eichmeier et al., 2016) [18]
	NC_001920.1	Grapevine yellow speckle viroid 1	0	17	0.41	32%	+ (Ward et al., 2011) [36]
	NC_015782.1	Grapevine Pinot gris virus	0	349	5.77E-3	74%	+ (Saldarelli et al., 2015) [2]
	NC_011535.1	Grapevine Algerian latent virus	0	68	0	12%	− (Tomitaka et al., 2016) [37]
	NC_003623.1	Grapevine fanleaf virus	0	104	0	18%	− (Eichmeier et al., 2010) [23]
	NC_003604.1	Grapevine virus A	0	85	0	11%	− (Minafra and Hadidi 2004) [38]
	GU733707.1	Grapevine virus B	0	149	0	3%	+ (Minafra and Hadidi 2004) [38]
	AY706994.1	G. rupestris vein feathering virus	0	371	0.03	13%	+ (Eichmeier et al., 2016) [18]
	NC_007448.1	Grapevine leafroll-associated virus 2	1768	1473	0.53	59%	+ (Lunden and Qiu 2012) [39]
	NC_004667.1	Grapevine leafroll-associated virus 3	2032	5479	1.29	79%	+ (Osman et Rowhani, 2006) [40]
AL_42	NC_016509.1	Grapevine leafroll-associated virus 1	0	489	0	9%	− (Kominek et al., 2005) [32], − (Gambino a Gribaudo 2006) [33]
	NC_003347.1	Grapevine fleck virus	66	348	0.31	53%	+ (Sabanadzovic et al., 1996) [34]
	NC_001948.1	G. rupestris stem pitting-associated virus	92	216	0.06	19%	+ (Terlizzi et al., 2011) [35]
	NC_001351.1	Hop stunt viroid	0	67	0.3	54%	+ (Eichmeier et al., 2016) [18]
	NC_001920.1	Grapevine yellow speckle viroid 1	0	24	1.9	64%	+ (Ward et al., 2011) [36]
	NC_015782.1	Grapevine Pinot gris virus	0	549	2.89E-3	89%	+ (Saldarelli et al., 2015) [2]
	NC_011535.1	Grapevine Algerian latent virus	0	27	0	6%	− (Tomitaka et al., 2016) [37]
	NC_003623.1	Grapevine fanleaf virus	0	107	0	14%	− (Eichmeier et al., 2010) [23]
	NC_003604.1	Grapevine virus A	0	58	0	8%	− (Minafra and Hadidi 2004) [38]
	GU733707.1	Grapevine virus B	0	105	0	1%	− (Minafra and Hadidi 2004) [38]
	AY706994.1	G. rupestris vein feathering virus	0	228	0.07	8%	− (Eichmeier et al., 2016) [18]
	NC_007448.1	Grapevine leafroll-associated virus 2	25	584	0.08	29%	+ (Lunden and Qiu 2012) [39]
	NC_004667.1	Grapevine leafroll-associated virus 3	327	2809	0.23	43%	+ (Osman et Rowhani, 2006) [40]

Table contains the list of reference sequences used for assembly, number of identified contigs, genome coverage, and RT-PCR detections with references. Red color indicates virus-positive samples. Column RT-PCR shows the positive (+) or negative (−) detection of the virus according to the listed references.

## Supplementary Materials

The following are available online at https://www.mdpi.com/2073-4425/11/9/1110/s1, Table S1: IDs show the details of clustering.

## References

[1] Giampetruzzi A., Roumi V., Roberto R., Malossini U., Yoshikawa N., La Notte P., Terlizzi F., Credi R., Saldarelli P. (2012). A new grapevine virus discovered by deep sequencing of virus- and viroid-derived small RNAs in Cv Pinot gris. Virus Res..

[2] Saldarelli P., Giampetruzzi A., Morelli M., Malossini U., Pirolo C., Bianchedi P., Gualandri V. (2015). Genetic variability of Grapevine Pinot gris virus and its association with grapevine leaf mottling and deformation. Phytopathology.

[3] Eichmeier A., Penazova E., Muljukina N. (2018). Survey of Grapevine Pinot gris virus in certified grapevine stocks in Ukraine. Eur. J. Plant. Pathol..

[4] Poojari S., Lowery T., Rott M., Schmidt A.M., Urbez-Torres J.R. (2016). First report of Grapevine Pinot gris virus in British Columbia, Canada. Plant. Dis..

[5] Eichmeier A., Penazova E., Nebish A. (2020). First Report of Grapevine Pinot gris virus on grapevines in Armenia. Plant. Dis..

[6] Wu Q., Habili N. (2017). The recent importation of Grapevine Pinot gris virus into Australia. Virus Genes.

[7] Fajardo T.V.M., Eiras M., Nickel O. (2017). First report of Grapevine Pinot gris virus infecting grapevine in Brazil. Australas. Plant Dis. Notes.

[8] Zamorano A., Medina G., Fernandez C., Cui W., Quiroga N., Fiore N. (2019). First report of Grapevine Pinot gris virus in grapevine in Chile. Plant. Dis..

[9] Rasool S., Naz S., Rowhani A., Golino D.A., Westrick N.M., Farrar K.D., Al Rwahnih M. (2017). First Report of Grapevine Pinot gris virus infecting grapevine in Pakistan. Plant Dis..

[10] Eichmeier A., Penazova E., Pavelkova R., Mynarzova Z., Saldarelli P. (2016). Detection of Grapevine Pinot gris girus in certified grapevine stocks in Moravia, Czech Republic. J. Plant Pathol..

[11] Bertazzon N., Filippin L., Forte V., Angelini E. (2016). Grapevine Pinot gris virus seems to have recently been introduced to vineyards in Veneto, Italy. Arch. Virol..

[12] Bertazzon N., Forte V., Filippin L., Causin R., Maixner M., Angelini E. (2017). Association between genetic variability and titre of Grapevine Pinot gris virus with disease symptoms. Plant Pathol..

[13] Malagnini V., de Lillo E., Saldarelli P., Beber R., Duso C., Raiola A., Zanotelli L., Valenzano D., Giampetruzzi A., Morelli M. (2016). Transmission of grapevine Pinot gris virus by *Colomerus vitis* (Acari: Eriophyidae) to grapevine. Arch. Virol..

[14] MADRP Agricultural Statistics‒B Series, Ministère de l’Agriculture, du développement Rural et de la Pêche. http://madrp.gov.dz/agriculture/statistiques-agricoles/.

[15] OIV (2019). Statistical Report on World Vitiviniculture.

[16] Hily J.M., Poulicard N., Candresse T., Vigne E., Beuve M., Renault L., Velt A., Spilmont A.S., Lemaire O. (2020). Datamining, Genetic diversity analyses, and phylogeographic reconstructions redefine the worldwide evolutionary history of Grapevine Pinot gris virus and Grapevine berry inner necrosis virus. Phytobiomes J..

[17] German S., Candresse T., Le Gall O., Lanneau M., Dunez J. (1992). Analysis of the dsRNAs of Apple chlorotic leaf-spot virus. J. Gen. Virol..

[18] Eichmeier A., Kominkova M., Kominek P., Baranek M. (2016). Comprehensive virus detection using next generation sequencing in grapevine vascular tissues of plants obtained from the wine regions of Bohemia and Moravia (Czech Republic). PLoS ONE.

[19] Glasa M., Predajna L., Kominek P., Nagyova A., Candresse T., Olmos A. (2014). Molecular characterization of divergent grapevine Pinot gris virus isolates and their detection in Slovak and Czech grapevines. Arch. Virol..

[20] Lehad A., Selmi I., Louanchi M., Aitouada M., Mahfoudhi N. (2019). Occurrence and diversity of Grapevine leafroll-associated virus 1 in Algeria. Phytopathol. Mediterr..

[21] Lehad A., Selmi I., Louanchi M., Aitouada M., Mahfoudhi N. (2015). Survey and genetic diversity of grapevine leafroll associated virus 2 in Algeria. Int. J. Phytopathol..

[22] Lehad A., Selmi I., Louanchi M., Aitouada M., Mahfoudhi N. (2015). Genetic Diversity of Grapevine leafroll-associated virus 3 in Algeria. J. Plant. Pathol..

[23] Eichmeier A., Baranek M., Pidra M. (2010). Analysis of genetic diversity and phylogeny of partial coat protein domain in Czech and Italian GFLV isolates. Plant. Prot. Sci..

[24] Eichmeier A., Pieczonka K., Penazova E., Pecenka J., Gajewski Z. (2017). Occurrence of Grapevine Pinot gris virus in Poland and description of asymptomatic exhibitions in grapevines. J. Plant. Dis. Prot..

[25] Eichmeier A., Kiss T., Penazova E., Pecenka J., Berraf-Tebbal A., Baranek M., Pokluda R., Cechova J., Gramaje D., Grzebelus D. (2019). MicroRNAs in *Vitis vinifera* cv. Chardonnay are differentially expressed in response to *Diaporthe* species. Genes.

[26] Andrews S. (2010). FastQC: A quality control tool for high throughput sequence data. http://www.bioinformatics.babraham.ac.uk/projects/fastqc.

[27] Zerbino D.R., Birney E. (2008). Velvet: Algorithms for de novo short read assembly using de Bruijn graphs. Genome Res..

[28] Eichmeier A., Kominkova M., Pecenka J., Kominek P. (2019). High-throughput small RNA sequencing for evaluation of grapevine sanitation efficacy. J. Virol. Methods.

[29] Tamura K., Nei M. (1993). Estimation of the number of nucleotide substitutions in the control region of mitochondrial-DNA in humans and chimpanzees. Mol. Biol. Evol..

[30] Kumar S., Stecher G., Tamura K. (2016). MEGA7: Molecular evolutionary genetics analysis version 7.0 for bigger datasets. Mol. Biol. Evol..

[31] Edgar R.C. (2004). MUSCLE: Multiple sequence alignment with high accuracy and high throughput. Nucleic Acids Res..

[32] Kominek P., Bryxiova A. (2005). Comparison of three techniques for detection of grapevine leafroll-associated virus 1. Acta Virol..

[33] Gambino G., Gribaudo I. (2006). Simultaneous detection of nine grapevine viruses by multiplex reverse transcription-polymerase chain reaction with coamplification of a plant RNA as internal control. Phytopathology.

[34] Sabanadzovic S., Saldarelli P., Savino V. (1996). Molecular diagnosis of grapevine fleck virus. Vitis.

[35] Terlizzi F., Li C., Ratti C., Qiu W., Credi R., Meng B. (2011). Detection of multiple sequence variants of Grapevine rupestris stem pitting-associated virus using primers targeting the polymerase domain and partial genome sequencing of a novel variant. Ann. Appl. Biol..

[36] Ward L.I., Burnip G.M., Liefting L.W., Harper S.J., Clover G.R.G. (2011). First report of Grapevine yellow speckle viroid 1 and Hop stunt viroid in Grapevine (*Vitis vinifera*) in New Zealand. Plant. Dis..

[37] Tomitaka Y., Usugi T., Fukami M., Tsuda S. (2016). First report of Grapevine Algerian latent virus infection on *Alstroemeria* spp. in Japan. Plant. Dis..

[38] Minafra A., Hadidi A. (1994). Sensitive detection of Grapevine Virus-A, Virus-B, or Leafroll-Associated-III from viruliferous mealybugs and infected tissue by cDNA amplification. J. Virol. Methods.

[39] Lunden S., Qiu W. (2012). First Report of Grapevine leafroll-associated virus 2 in a hybrid grape ’Vidal Blanc’ in Missouri. Plant. Dis..

[40] Osman F., Rowhani A. (2006). Application of a spotting sample preparation technique for the detection of pathogens in woody plants by RT-PCR and real-time PCR (TaqMan). J. Virol. Methods.

[41] Buoso S., Pagliari L., Musetti R., Fornasier F., Martini M., Loschi A., Fontanella M.C., Ermacora P. (2020). With or without you: Altered plant response to boron-deficiency in hydroponically grown grapevines infected by Grapevine Pinot gris virus suggests a relation between grapevine leaf mottling and deformation symptom occurrence and boron plant availability. Front. Plant. Sci..

[42] Bianchi G.L., De Amicis F., De Sabbata L., Di Bernardo N., Governatori G., Notino F. (2015). Occurrence of Grapevine Pinot gris virus in Friuli Venezia Giulia (Italy): Field monitoring and virus quantification by real-time RT-PCR. EPPO Bull..

[43] Tarquini G., Ermacora P., Bianchi G.L., De Amicis F., Pagliari L., Martini M., Loschi A., Saldarelli P., Loi N., Musetti R. (2018). Localization and subcellular association of Grapevine Pinot gris virus in grapevine leaf tissues. Protoplasma.

[44] Al Rwahnih M., Golino D., Rowhani A. (2016). First report of Grapevine Pinot gris virus infecting grapevine in the United States. Plant. Dis..

[45] Tarquini G., De Amicis F., Martini M., Ermacora P., Loi N., Musetti R., Bianchi G.L., Firrao G. (2019). Analysis of new grapevine Pinot gris virus (GPGV) isolates from Northeast Italy provides clues to track the evolution of a newly emerging clade. Arch. Virol..

