# Supplementary material for: Survey and Diversity of Grapevine Pinot gris virus in Algeria and Comprehensive High-Throughput Small RNA Sequencing Analysis of Two Isolates from Vitis vinifera cv. Sabel Revealing High Viral Diversity
Source: Genes (Basel). 2020 Sep 22;11(9):1110. doi: 10.3390/genes11091110 (PMC7563602; doi:10.3390/genes11091110)
Supplement: Supplementary file 1 [file genes-11-01110-s001.pdf]

Supplementary Table 1, IDs shown the details of clustering.

AncId, Desc1, Desc2, Branch Length 1, Branch Length 2

65, Boumerdes_41, Boumerdes_42, 0.0060263800, 0.0060354000

64, 65, Boumerdes_36, 0.0029957500, 0.0120690000

66, Boumerdes_31, Boumerdes_32, 0.0090308700, 0.0029979200

67, 64, 66, 0.0000000000, 0.0000000000

68, 67, Medea_11, 0.0000000000, 0.0151077000

69, 68, 101-144923_rootstock_clone_Ukraine, 0.0030077700, 0.0030077700

72, 69, Alger_53, 0.0029961300, 0.0150796000

74, 72, RC-5145_Rcatiteli_Ukraine, 0.0000000000, 0.0029902200

70, 41B-3721_rootstock_clone_Ukraine, 55_Poland, 0.0151350000, 0.0121770000

71, CS-143141_Cabernet_Sauvignon_Ukraine, 56_Poland, 0.0120842000, 0.0029881900

73, 70, 71, 0.0029269900, 0.0030012200

75, 74, 73, 0.0000000000, 0.0000000000

62, R-13101_Riesling_Ukraine, R-6846_Riesling_Ukraine, 0.0029854200, 0.0060271100

63, R-2071_Riesling_Ukraine, 62, 0.0121532000, 0.0029353100

76, 75, 63, 0.0000000000, 0.0091339400

86, 48_Poland, 49_Poland, 0.0000000000, 0.0030106800

78, 12_Poland, 45_Poland, 0.0000000000, 0.0090785500

79, 41_Poland, 44_Poland, 0.0030096500, 0.0000000000

80, 13_Poland, 79, 0.0000000000, 0.0000000000

81, 78, 80, 0.0030096500, 0.0000000000

85, 86, 81, 0.0000000000, 0.0030025100

84, 43_Poland, 85, 0.0121746000, 0.0000000000

83, 47_Poland, 84, 0.0029990600, 0.0000000000

82, 46_Poland, 83, 0.0030029300, 0.0000000000

77, 76, 82, 0.0060070300, 0.0030189800

61, 77, 42_Poland, 0.0127290000, 0.0313408000

55, ZA505-3N_Italy, ZA505-1N_Italy, 0.0000000000, 0.0213086000

57, 55, ZA505-2N_Italy, 0.0000000000, 0.0030034700

59, 57, SK30_Slovakia, 0.0000000000, 0.0090659600

58, 59, RC-6054_Rcatiteli_Ukraine, 0.0030095000, 0.0060310100

51, SK01_Slovakia, SK13_Slovakia, 0.0090502400, 0.0000000000

52, 51, MOLA3x3_Italy, 0.0000000000, 0.0090456100

53, 52, CS-22103_Cabernet_Sauvignon_Ukraine, 0.0000000000, 0.0060174000

45, ZA505-9A_Italy, ZA505-2A_Italy, 0.0000000000, 0.0000000000

46, ZA505-3A_Italy, 45, 0.0000000000, 0.0000000000

47, ZA505-5A_Italy, 46, 0.0000000000, 0.0000000000

48, ZA505-8A_Italy, 47, 0.0030061100, 0.0000000000

49, BE(FA59)1A_Italy, 48, 0.0000000000, 0.0030026500

50, 5BB-9191_rootstock_clone_Ukraine, 49, 0.0030414200, 0.0060333600

54, 53, 50, 0.0000000000, 0.0029937900

56, 58, 54, 0.0030033100, 0.0000000000

60, Boumerdes_33, 56, 0.0000000000, 0.0121234000

87, 61, 60, 0.3683410000, 0.3689190000
